# Relationship between hemodynamic parameters and severity of ischemia-induced left ventricular wall thickening during cardiopulmonary resuscitation of consistent quality

**DOI:** 10.1371/journal.pone.0208140

**Published:** 2018-11-28

**Authors:** Se-hyeok Park, Yong Deok Lim, Yong Hun Jung, Kyung Woon Jeung

**Affiliations:** Department of Emergency Medicine, Chonnam National University Hospital, Gwangju, Republic of Korea; Azienda Ospedaliero Universitaria Careggi, ITALY

## Abstract

Ischemia-induced left ventricular (LV) wall thickening compromises the hemodynamic effectiveness of cardiopulmonary resuscitation (CPR). However, accurate assessment of the severity of ischemia-induced LV wall thickening during CPR is challenging. We investigated, in a swine model, whether hemodynamic parameters, including end-tidal carbon dioxide (ETCO_2_) level, are linearly associated with the severity of ischemia-induced LV wall thickening during CPR of consistent quality. We retrospectively analyzed 96 datasets for ETCO_2_ level, arterial pressure, LV wall thickness, and the percent of measured end-diastolic volume (%EDV) relative to EDV at the onset of ventricular fibrillation from eight pigs. Animals underwent advanced cardiovascular life support based on resuscitation guidelines. During CPR, LV wall thickness progressively increased while %EDV progressively decreased. Systolic and diastolic arterial pressure and ETCO_2_ level were significantly correlated with LV wall thickness and %EDV. Linear mixed effect models revealed that, after adjustment for significant covariates, systolic and diastolic arterial pressure were not associated with LV wall thickness or %EDV. ETCO_2_ level had a significant linear relationship with %EDV (*P* = 0.004). However, it could explain only 28.2% of the total variance of %EDV in our model. In conclusion, none of the hemodynamic parameters examined in this study appeared to provide sufficient information on the severity of ischemia-induced LV wall thickening.

## Introduction

More than 50 years have passed since the introduction of modern cardiopulmonary resuscitation (CPR) [1]. A tremendous number of studies have been performed to enhance understanding of cardiac arrest and CPR. However, many aspects of cardiac arrest pathophysiology remain to be determined.

CPR is performed to generate enough blood flow to sustain vital organs until restoration of spontaneous circulation (ROSC). Several studies have reported that progressive left ventricular (LV) wall thickening occurs during CPR [2–6]. This ischemia-induced LV wall thickening inevitably results in a reduction in the LV chamber volume [2,3]. Thus, the volume of blood ejected by chest compression decreases as the ischemia-induced LV wall thickening progresses, ultimately precluding successful resuscitation [2,3,7]. In experimental settings, echocardiographic assessment with transesophageal or transmediastinal approaches has been used to observe this phenomenon, by monitoring LV wall thickness and chamber volume [2–6]. However, the echocardiographic assessments using these approaches are usually impractical or unavailable in clinical resuscitation settings. Transthoracic echocardiography is frequently available in clinical resuscitation settings. However, acquisition of echocardiographic images of adequate quality for the assessment of LV wall thickness and chamber volume requires considerable time, as the results of echocardiographic measurements vary widely with relatively minor changes in the transducer position. Thus, unlike the echocardiographic assessment with transesophageal or transmediastinal approaches in experimental settings, the acquisition of adequate quality images with transthoracic echocardiography during actual cardiac arrest resuscitation is challenging without causing excessive interruptions to CPR. Furthermore, information on patients’ pre-arrest cardiac dimensions should be considered in order to accurately assess the severity of ischemia-induced LV wall thickening, as cardiac dimensions vary widely among individuals [8]. However, this information is usually not available during actual CPR, making the assessment of ischemia-induced LV wall thickening during CPR more difficult.

Ischemia-induced LV wall thickening compromises the hemodynamic effectiveness of CPR by reducing the stroke volume generated by chest compression [2,3]. Several studies have suggested that hemodynamic parameters including arterial pressure and end-tidal carbon dioxide (ETCO_2_) level, which are used as physiological monitoring parameters during clinical CPR [9–11], correlate with blood flow generated during CPR [12–15]. Thus, these hemodynamic parameters may reflect the severity of ischemia-induced LV wall thickening during CPR. However, to our knowledge, no studies have evaluated whether these hemodynamic parameters can be used to estimate the severity of ischemia-induced LV wall thickening during CPR.

In the present study, we investigated, in a swine model of out-of-hospital cardiac arrest, whether hemodynamic parameters, including arterial pressure and ETCO_2_ level, are linearly associated with the severity of ischemia-induced LV wall thickening during CPR of consistent quality, and therefore can be utilized as a tool to estimate the severity of ischemia-induced LV wall thickening during advanced cardiovascular life support (ACLS) performed according to resuscitation guidelines. We hypothesized that arterial pressure and ETCO_2_ level would be linearly associated with the severity of ischemia-induced LV wall thickening during CPR.

## Materials and methods

We retrospectively analyzed data derived from a previous study investigating the effects of pralidoxime administered during CPR on ischemia-induced LV wall thickening in 16 Yorkshire/Landrace cross pigs weighing 25.2 ± 2.9 kg. Several studies indicated that 2,3-butanedione monoxime attenuated ischemia-induced LV wall thickening [5,6]. Both pralidoxime and 2,3-butanedione monoxime belong to the same oxime family and share several common mechanisms of action. Thus, in the previous study, we hypothesized that pralidoxime would also reverse ischemia-induced LV wall thickening in a similar manner to 2,3-butanedione monoxime. However, unlike 2,3-butanedione monoxime, pralidoxime had no effect on ischemia-induced LV wall thickening, but significantly improved aortic pressure and coronary perfusion pressure (CPP) in the study. In the present study, data from eight animals that received only standard ACLS in the study were included. Animal care and experiments were in accord with the National Institutes of Health Guide for the Care and Use of Laboratory Animals. The experimental protocol was approved by the Animal Care and Use Committee of Chonnam National University (CNU IACUC-H-2017-18).

### Animal preparation

Animal preparation techniques have been described in detail previously [5,6]. Animals were orally intubated following intramuscular injection of ketamine (20 mg/kg) and xylazine (2.2 mg/kg). Thereafter, animals were mechanically ventilated with a tidal volume of 10 ml/kg and a respiratory rate adjusted to achieve normocapnia. The following procedures were performed under general anesthesia using 70%:30% N_2_O:O_2_ and 0.5%–2% sevoflurane. A 7 F double-lumen catheter was inserted via the left femoral artery for arterial pressure monitoring and blood sampling, and a 7 F introducer sheath was inserted via the right external jugular vein for right atrial pressure monitoring and right ventricular pacing wire insertion. To obtain echocardiographic images of adequate quality for the assessment of LV wall thickness and chamber volume during CPR, a skin incision was made immediately below the xiphoid process and a pocket extending 4–5 cm was made under the sternum. A transesophageal echocardiography probe (UST-5293-5; Hitachi Aloka Medical Ltd., Tokyo, Japan) was precordially inserted via the pocket and the best obtainable long-axis view of the LV was sought using the probe manipulation [5,6]. In our previous studies, in which transthoracic or transesophageal approach was used for echocardiographic imaging during CPR, chest compressions precluded acquisition of echocardiographic images of adequate quality. However, this method enabled acquisition of adequate quality images without interruption of chest compression. An ETCO_2_ sample line (B40 Patient Monitor; GE Healthcare, Chalfont St. Giles, UK) was attached to the rostral end of the endotracheal tube.

### Experimental protocol

**Fig 1:**
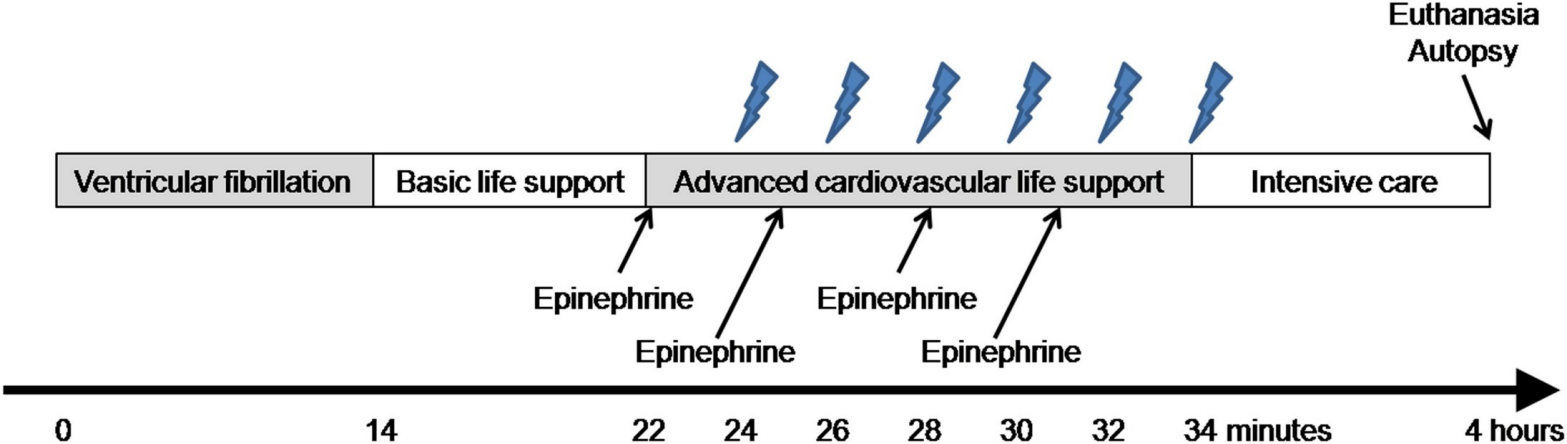
Experimental timeline. Lightning marks indicate the onset of a 10-second pause in chest compressions for rhythm analysis and a 150-J shock, if indicated.

The experimental timeline is shown in Fig 1. Ventricular fibrillation (VF) was induced by delivering an electrical current (60 Hz and 30 mA alternating current) via a right ventricular pacing wire and mechanical ventilation was discontinued. After 14 minutes of untreated VF, cycles of 30 chest compressions followed by two ventilations with ambient air were provided to simulate basic life support (BLS). After eight minutes of simulated BLS, ACLS was started based on recent resuscitation guidelines [11]. Chest compressions were delivered at a rate of 100/min and a depth of 20% of the anterior-posterior diameter of the chest using a mechanical chest compression device (Life-Stat; Michigan Instruments, Grand Rapids, MI, USA). Asynchronous positive-pressure ventilations were provided with high-flow oxygen (14 l/min) at a rate of 10/min using a volume-marked bag devised by Cho et al. [16]. In order to deliver a constant volume of approximately 250 ml, the investigator ventilating the animals placed his thumb and middle finger on designated positions on the surface of the bag, and squeezed the bag, touching the middle finger to the thumb slightly. Cho et al. reported that this method delivered the desired tidal volume with regularity and precision [16]. The investigator was blinded to the arterial pressure and ETCO_2_ level. During ACLS, epinephrine (0.02 mg/kg) was administered every three minutes, and defibrillation using a single biphasic 150-J electric shock was attempted at two-minute intervals, if indicated. If ROSC was not achieved within 12 minutes of ACLS, resuscitation efforts were discontinued. All animals included in the present study failed to achieve ROSC, and thus an additional procedure for euthanasia was not applied.

### Measurements

Arterial pressure was continuously monitored (CS/3 CCM;Datex-Ohmeda, Helsinki, Finland) and transferred to a personal computer using S/5 Collect software (Datex-Ohmeda, Helsinki, Finland). Systolic arterial pressure was defined as the peak arterial pressure during the chest compression phase, while diastolic arterial pressure was defined as the lowest inflection point at the beginning of the next compression-induced pressure upstroke. CPP was calculated by subtracting the right atrial end-diastolic pressure from time-coincident diastolic arterial pressure. Arterial pressure and CPP were calculated by averaging pressures from five consecutive compressions at two-minute intervals during BLS and at one-minute intervals during ACLS. Cumulative CPP during BLS was calculated as the sum of CPP values obtained during BLS. ETCO_2_ values were determined every one minute by averaging the ETCO2 values for the preceding 30-second interval. Echocardiograms were obtained by an experienced investigator five minutes before induction of VF, immediately after initiation of VF, and in one-minute intervals during ACLS. LV wall thickness and end-diastolic volume (EDV) during CPR were measured at the frame showing maximal chamber dimension of the LV following release of chest compression. LV wall thickness was measured in the lateral wall at the mid-ventricular level. EDV and ejection fraction were calculated using Simpson’s method. EDV, which was the primary outcome measurement of the present study, was expressed as a percent of measured EDV relative to EDV at the onset of VF (%EDV), to correct for its substantial inter-animal variation.

### Statistical analysis

Data were analyzed using the R language version 3.3.3 (R Foundation for Statistical Computing, Vienna, Austria) and T&F program version 2.2 (YooJin BioSoft, Goyang, Korea). Continuous variables were tested for normality through the Kolmogorov–Smirnov test. Normally distributed variables were expressed as mean ± standard deviation; non-normally distributed variables were reported as medians with interquartile ranges. Correlation between repeatedly measured variables was assessed with the rmcorr package [17]. Multiple linear mixed effect models were generated in order to evaluate the independent effects of each hemodynamic parameter on the response variables including LV wall thickness and %EDV. Baseline variables and cumulative CPP during BLS were independently analyzed regarding the significance of their effectiveness in explaining the response variables, and significant variables (*P* value cutoff = 0.05) were further used for covariate adjustment in the mixed effect models. Variables showing serious multicollinearity were removed from the model. Time and each hemodynamic parameter were used as fixed effect covariates with the random effect of intercept for subjects. A random slope was not used due to the significant correlation between random intercept and slope. Semi-partial R-squared values of the fixed effects were computed for hemodynamic parameters and time using the r2glmm package [18]. A two-tailed significance level of 0.05 was used for statistical significance.

## Results

**Fig 2:**
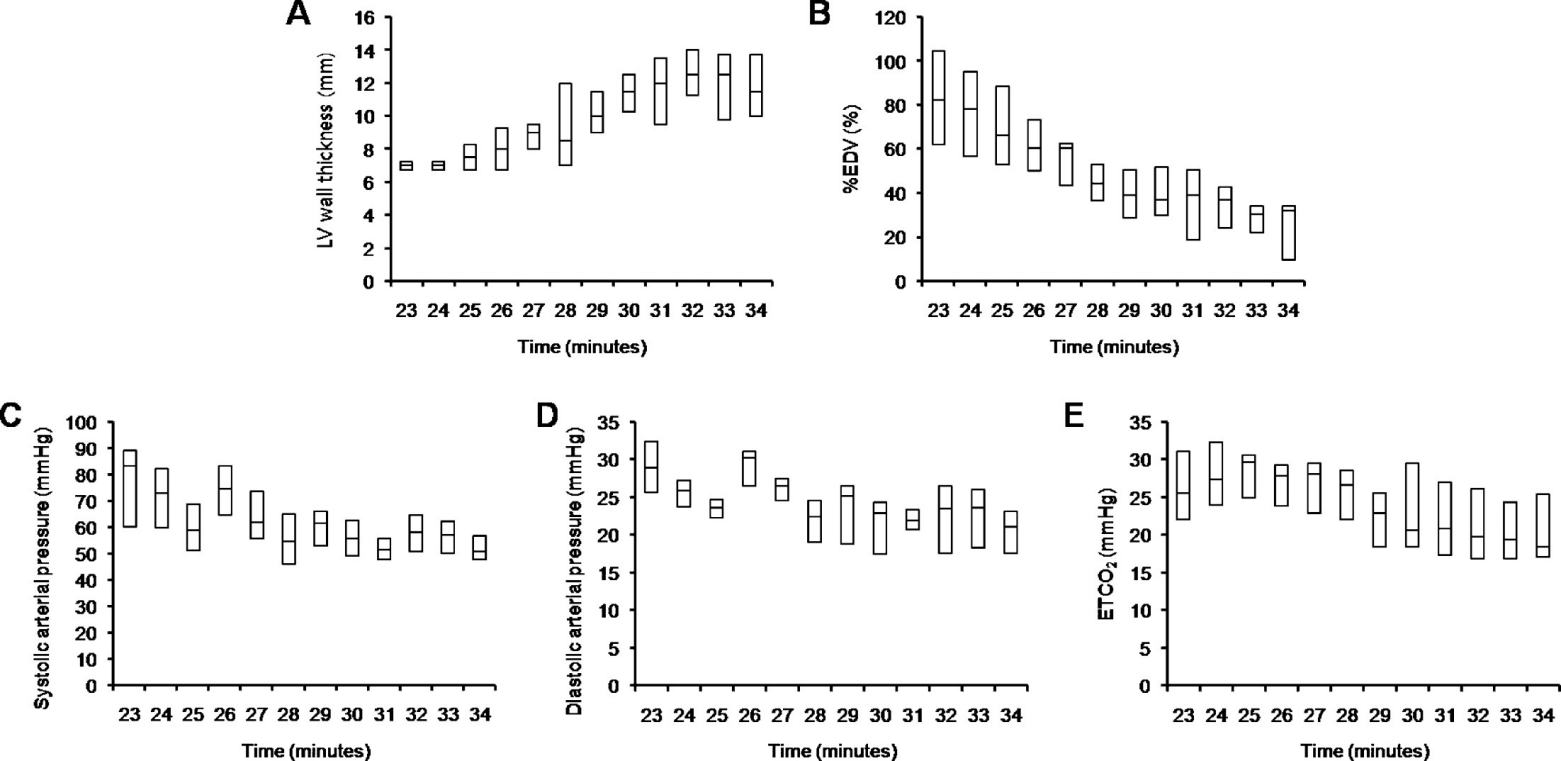
**LV wall thickness (A), %EDV (B), systolic arterial pressure (C), diastolic arterial pressure (D), and ETCO**_**2**_ **level (E) during cardiopulmonary resuscitation.** Data are presented as the median and interquartile ranges. LV, left ventricular; EDV, end-diastolic volume; ETCO_2_, end-tidal carbon dioxide.

Pre-arrest baseline measurements of included animals were within normal limits (Table 1). All animals underwent the 12 minute-ACLS period without achieving ROSC. Thus, a total of 96 sets of ETCO_2_ level, arterial pressure, LV wall thickness, and %EDV data were collected (12 times with one-minute intervals in eight animals) and included in the analyses. Fig 2 shows LV wall thickness, %EDV, systolic arterial pressure, diastolic arterial pressure, and ETCO_2_ level during CPR. During CPR, LV wall thickness progressively increased while %EDV progressively decreased. S1 Movie shows representative echocardiograms showing the progression of ischemia-induced LV wall thickening. Each repeatedly measured response variable, including LV wall thickness and %EDV, were significantly correlated with each hemodynamic parameter, including systolic and diastolic arterial pressure, and ETCO_2_ level (Table 2). Tables 3 and 4 show the fixed effects of time and each hemodynamic parameter on LV wall thickness and %EDV in linear mixed effect models, respectively. In the linear mixed effect models, the relationships between hemodynamic parameters and response variables were not significant after adjustment for significant covariates, except for the relationship between ETCO_2_ level and %EDV. There was a significant linear association between ETCO_2_ level and %EDV (*P* = 0.004). Throughout our models, time was the most significant factor associated with the response variables. For example, in the model including time and ETCO_2_, time explained 66.6% of the total variance of %EDV when adjusted for significant covariates (semi-partial R^2^ = 0.666; 95% confidence interval [CI], 0.571–0.750; *P* < 0.001), while ETCO_2_ level explained only 28.2% of the total variance of %EDV (semi-partial R^2^ = 0.282; 95% CI = 0.149–0.428, *P* = 0.004). The results were also similar in the model including all three hemodynamic parameters (Tables 5 and 6).

**Table 1:** Pre-arrest baseline measurements and coronary perfusion pressure during basic life support.

Weight (kg)	24.1 (23.7–24.9)
Baseline systolic arterial pressure (mmHg)	116 (108–123)
Baseline diastolic arterial pressure (mmHg)	80 (67–85)
Baseline mean arterial pressure (mmHg)	93 (84–100)
Baseline systolic right atrial pressure (mmHg)	14 (12–15)
Baseline diastolic right atrial pressure (mmHg)	9 (8–10)
Baseline mean right atrial pressure (mmHg)	12 (10–13)
Baseline heart rate (beats/min)	92 (79–97)
Baseline ETCO_2_ (mmHg)	34.5 (32.3–35.8)
Baseline pH	7.498 (7.462–7.543)
Baseline PaCO_2_ (mmHg)	35.4 (31.8–39.3)
Baseline PaO_2_ (mmHg)	151.7 (142.7–157.1)
Baseline base excess (mmol/l)	4.4 (1.7–5.8)
Baseline HCO_3_^-^ (mmol/l)	26.6 (25.5–29.1)
Baseline SaO_2_ (%)	99.6 (99.0–99.9)
Baseline troponin (ng/ml)	0.206 (0.188–0.244)
Baseline lactate (mmol/l)	1.3 (0.7–1.8)
Baseline left ventricular ejection fraction (%)	50.3 (45.1–54.2)
Cumulative CPP during BLS (mmHg)	16.6 (11.0–19.8)

Data are presented as medians with interquartile ranges. ETCO_2_, end-tidal carbon dioxide; PaCO_2_, partial pressure of carbon dioxide; PaO_2_, partial pressure of oxygen, HCO_3_^-^, bicarbonate, SaO_2_, oxygen saturation; CPP, coronary perfusion pressure; BLS, basic life support.

**Table 2:** Correlations between repeatedly measured variables.

Variables	Correlation coefficient	95% CI	*P*
LV wall thickness			
ETCO_2_	-0.55	-0.681 –-0.383	<0.001
Systolic arterial pressure	-0.476	-0.624 –-0.295	<0.001
Diastolic arterial pressure	-0.477	-0.625 –-0.297	<0.001
%EDV			
ETCO_2_	0.303	0.098–0.483	0.004
Systolic arterial pressure	0.405	0.212–0.567	<0.001
Diastolic arterial pressure	0.437	0.250–0.593	<0.001

CI, confidence interval; LV, left ventricular; ETCO_2_, end-tidal carbon dioxide; EDV, end-diastolic volume.

**Table 3:** Fixed effects of time and hemodynamic parameters on LV wall thickness in mixed effect models.

	Coefficient	SE	*P*	Semi-partial R^2^	95% CI
Time	0.514	0.044	<0.001	0.581	0.469–0.684
ETCO_2_ (mmHg)	-0.069	0.043	0.110	0.109	0.021–0.244
Time*	0.543	0.042	<0.001	0.635	0.534–0.726
ETCO_2_ (mmHg)*	-0.018	0.034	0.605	0.006	0–0.075
Time	0.546	0.045	<0.001	0.590	0.479–0.691
Systolic arterial pressure (mmHg)	-0.004	0.014	0.782	0.002	0–0.060
Time*	0.554	0.043	<0.001	0.633	0.530–0.724
Systolic arterial pressure (mmHg)*	0.001	0.012	0.937	0	0–0.052
Time	0.525	0.045	<0.001	0.565	0.449–0.671
Diastolic arterial pressure (mmHg)	-0.049	0.042	0.248	0.023	0–0.115
Time*	0.536	0.045	<0.001	0.585	0.473–0.687
Diastolic arterial pressure (mmHg)*	-0.030	0.042	0.469	0.006	0–0.073

* Baseline variables used to adjust the mixed effect model included heart rate, SaO_2_, and troponin. SE, standard error; CI, confidence interval; ETCO_2_, end-tidal carbon dioxide.

**Table 4:** Fixed effects of time and hemodynamic parameters on %EDV in mixed effect models.

	Coefficient	SE	*P*	Semi-partial R^2^	95% CI
Time	-5.512	0.402	<0.001	0.654	0.557–0.741
ETCO_2_ (mmHg)	-1.239	0.399	0.003	0.318	0.182–0.461
Time*	-5.448	0.395	<0.001	0.666	0.571–0.750
ETCO_2_ (mmHg)*	-1.122	0.372	0.004	0.282	0.149–0.428
Time	-5.032	0.424	<0.001	0.578	0.465–0.681
Systolic arterial pressure (mmHg)	-0.114	0.129	0.378	0.019	0–0.108
Time*	-4.967	0.419	<0.001	0.591	0.480–0.691
Systolic arterial pressure (mmHg)*	-0.077	0.124	0.533	0.009	0–0.084
Time	-4.719	0.421	<0.001	0.536	0.415–0.647
Diastolic arterial pressure (mmHg)	0.198	0.395	0.617	0.004	0–0.069
Time*	-4.713	0.420	<0.001	0.545	0.426–0.655
Diastolic arterial pressure (mmHg)*	0.210	0.391	0.592	0.004	0–0.068

* Baseline troponin level was used to adjust the mixed effect model. SE, standard error; CI, confidence interval; ETCO_2_, end-tidal carbon dioxide.

**Table 5:** Fixed effects of time, systolic arterial pressure, diastolic arterial pressure, and ETCO_2_ level on LV wall thickness in mixed effect model including all these hemodynamic parameters.

	Coefficient	SE	*P*	Semi-partial R^2^	95% CI
Time	0.530	0.047	<0.001	0.588	0.476–0.689
ETCO_2_ (mmHg)	-0.022	0.031	0.498	0.012	0–0.092
Systolic arterial pressure (mmHg)	0.008	0.014	0.561	0.007	0–0.076
Diastolic arterial pressure (mmHg)	-0.045	0.048	0.351	0.009	0–0.084

Baseline variables used to adjust the mixed effect model included heart rate, SaO_2_, and troponin. SE, standard error; CI, confidence interval; ETCO_2_, end-tidal carbon dioxide.

**Table 6:** Fixed effects of time, systolic arterial pressure, diastolic arterial pressure, and ETCO_2_ level on %EDV in mixed effect model including all these hemodynamic parameters.

	Coefficient	SE	*P*	Semi-partial R^2^	95% CI
Time	-5.342	0.444	<0.001	0.621	0.516–0.715
ETCO_2_ (mmHg)	-1.167	0.415	0.007	0.284	0.150–0.429
Systolic arterial pressure (mmHg)	0.039	0.158	0.803	0.002	0–0.059
Diastolic arterial pressure (mmHg)	0.108	0.452	0.811	0.001	0–0.055

Baseline troponin level was used to adjust the mixed effect model. SE, standard error; CI, confidence interval; ETCO_2_, end-tidal carbon dioxide.

## Discussion

To our knowledge, this is the first study to assess hemodynamic parameters as potential tools to estimate the severity of ischemia-induced LV wall thickening during CPR. Our results indicated that systolic arterial pressure and diastolic arterial pressure were not associated with the severity of ischemia-induced LV wall thickening. Although ETCO_2_ level had a significant linear association with the %EDV, its ability to explain the variance of %EDV was very limited.

Ischemia-induced LV wall thickening during CPR has previously been regarded as a manifestation of myocardial ischemic contracture [2–6]. However, a recent study suggests that the ischemia-induced LV wall thickening that occurs early during CPR, as observed in the present study, is different from that from myocardial ischemic contracture [19]. Myocardial ischemic contracture typically occurs in a more delayed fashion, and its onset is associated with depletion of myocardial adenosine triphosphate to less than 10% of normal baseline levels [20,21]. Ayoub et al. reported that LV wall thickening during CPR occurred with myocardial adenosine triphosphate levels of approximately 55% of pre-arrest baseline levels after 13 minutes of VF in a pig model of cardiac arrest [19]. Although the myocardial adenosine triphosphate level was not measured in our study, the LV wall thickening in the present study was likely a different phenomenon from myocardial ischemic contracture given its timing of onset. Several studies have suggested that LV wall thickening that occurs early during CPR, in contrast to ischemic contracture which is known as an irreversible state, can be an object of treatment and that therapeutic interventions reversing this phenomenon facilitate successful resuscitation by improving the hemodynamic effectiveness of CPR [4–6,22]. Lee et al. reported that 2,3-butanedione monoxime attenuated ischemia-induced LV wall thickening and improved resuscitability in a pig model of cardiac arrest [6]. Ayoub et al. reported in a pig model of cardiac arrest that cariporide, a selective sodium-hydrogen exchanger isoform-1 inhibitor, attenuated ischemia-induced LV wall thickening, maintained CPP above a threshold level that renders ROSC likely, and improved resuscitability [4].

ETCO_2_ is a simple, noninvasive, and easily-applicable parameter that correlates closely with the blood flow generated during CPR [12,13]. Therefore, we initially hypothesized that ETCO_2_ level would be able to track changes in the %EDV caused by ischemia-induced LV wall thickening, under conditions of consistent chest compression and ventilation. Consistent with our hypothesis, ETCO_2_ level had a significant linear relationship with %EDV. However, although CPR was conducted under consistent compression and ventilation settings in the present study, the correlation coefficient and semi-partial R-squared values were not high enough to be clinically useful. The animals were subjected to the same duration of untreated VF in the present study. A previous study reported that the severity of ischemia-induced LV wall thickening was proportional to no-flow time (period from the onset of cardiac arrest to the start of CPR) [2]. The use of the same duration of untreated VF in the present study might have resulted in reduced variability in the severity of ischemia-induced LV wall thickening, and this reduced variability might have attenuated the correlation between ETCO_2_ level and %EDV. On the other hand, the low correlation coefficient and semi-partial R-squared values may indicate that other factors, besides cardiac output, affected the level of ETCO_2_ in the present study. Morimoto et al. observed ETCO_2_ level while maintaining a constant cardiac output during open-chest CPR in nine mongrel dogs, and reported that the ETCO_2_ level changed, despite constant cardiac output, probably due to changes in several factors, including alveolar dead space and pulmonary vascular tone [23]. In the present study, the quality of CPR, which is an important determinant of ETCO_2_ level during CPR [24], was consistent throughout the process, which is similar to CPR using a mechanical CPR device. However, in clinical resuscitation settings, where manual chest compressions and ventilations are provided, it may be difficult to maintain consistent quality of CPR. The variation in CPR quality, frequently encountered during manual CPR, would further limit the clinical utility of ETCO_2_ level in estimating the severity of ischemia-induced LV wall thickening.

Unlike ETCO_2_ level, neither the systolic nor the diastolic arterial pressure was associated with the %EDV in the present study. This may be due to the fact that, in contrast to ETCO_2_ level, arterial pressure depends not only on cardiac output, but more critically on vascular tone. Another possible explanation for this finding is related to the mechanism of blood flow during CPR. There are two main theories explaining the mechanism of blood flow during CPR: the cardiac pump theory and the thoracic pump theory [25–28]. The cardiac pump theory postulates that direct cardiac compression is responsible for the forward blood flow during CPR, while the thoracic pump theory postulates that changes in intrathoracic pressure induced by external chest compressions produce forward blood flow and arterial pressure fluctuations. In the thoracic pump theory, the heart acts as a passive conduit rather than a pump. The ischemia-induced LV wall thickening would theoretically compromise the cardiac pump, but it would not affect the thoracic pump mechanism. Thus, this finding, as well as the low semi-partial R-squared values of ETCO_2_ level, may be attributed to the existence of the thoracic pump mechanism.

None of the hemodynamic parameters examined in the present study appeared to provide sufficient information on the severity of ischemia-induced LV wall thickening. In addition, time was the most significant factor associated with the severity of ischemia-induced LV wall thickening in the present study. This finding is in agreement with previous observations that ischemia-induced LV wall thickening is a time-dependent progressive process, rather than an all-or-nothing process [2,5,6]. Our results, together with these previous studies, suggest that elapsed time since the onset of cardiac arrest, rather than the hemodynamic parameters during CPR, may be useful in estimating the severity of ischemia-induced LV wall thickening. However, our study could not specify the elapsed time since the onset of cardiac arrest when the ischemia-induced LV wall thickening became severe enough to hamper successful resuscitation, and thus the therapeutic interventions reversing this phenomenon became beneficial. Further studies are required to address this issue.

Our study has several important limitations. First, data were acquired from young healthy pigs. Thus, caution is required with regard to direct extrapolation to humans. Second, our study analyzed serial hemodynamic and echocardiographic data acquired from a relatively small number of animals. Although power analysis revealed a power of 85.9% (95% CI, 83.6–88.0) to detect the effect of ETCO_2_ level on %EDV at α = 0.05, further studies including a larger number of animals are required to confirm our findings. Third, all animals included in the present study could not achieve ROSC. Previous studies have suggested that the hemodynamic parameters, as well as the severity of ischemia-induced LV wall thickening, differ significantly between subjects with and without ROSC [7,12,14,29]. Thus, the relationship between hemodynamic parameters and severity of ischemia-induced LV wall thickening may also differ between subjects with and without ROSC. Fourth, the present study was conducted in a well-controlled experimental setting. Studies indicate that a number of subject and resuscitation factors, including arrest etiology and initial cardiac rhythm, influence the ETCO_2_ level [30,31]. Thus, variation in these factors, which is routinely encountered in clinical resuscitation settings, may result in different outcomes. Fifth, echocardiograms were conducted by a single investigator. Although the investigator had substantial experience of the echocardiographic technique used in the present study, observer-dependent bias might have occurred. Sixth, we could not identify factors related to the severity of ischemia-induced LV wall thickening.

## Conclusions

In conclusion, the present study, which investigated whether hemodynamic parameters can be used to estimate the severity of ischemia-induced LV wall thickening during CPR of consistent quality, showed that systolic and diastolic arterial pressure were not associated with the severity of ischemia-induced LV wall thickening. While ETCO_2_ level had a significant linear relationship with the severity of ischemia-induced LV wall thickening, it explained only a small proportion of the variance of its severity.

## Supporting information

S1 Movie Representative echocardiograms showing the progression of ischemia-induced LV wall thickening. Echocardiograms were obtained at pre-arrest baseline (left), 16 minutes after the onset of ventricular fibrillation (middle), and 24 minutes after the onset of ventricular fibrillation (right). VF, ventricular fibrillation; BLS, basic life support; ACLS, advanced cardiovascular life support.
(AVI)

S1 Data Raw data. (XLSX)

S1 File ARRIVE guidelines checklist. (DOC)
